# Matter: Living and Dead

**Published:** 1871-04

**Authors:** I. N. Danforth

**Affiliations:** Chicago


### Chicago Medical Journal.

A MONTHLY RECORD OF

*Medicine, Surgery, and the Collateral Sciences.*

EDITED BY J. ADAMS ALLEN, M.D., LL.D.; AND WALTER HAY, M.D.

VOL. XXVIII.—APRIL, 1871.—No. 4.

### Original Communications.

ARTICLE I.—*Matter: Living and Dead.* By I. N. DANFORTH, M.D., Chicago.

The views of Prof. Beale, now so widely disseminated, are doubtless bearing excellent fruit, and producing excellent results; and yet I believe it is quite possible that some erroneous conclusions may be drawn from his published writings, if his language is always to be literally understood. In fact, if we always strictly construe his sentences, and take him at his word, we can scarcely help looking upon ourselves as more dead than alive; as fitter subjects for the charnel-house, than for the congregations of *living* men. The proposition that "nothing that lives is alive in every part"* is demonstrably true in the latitudinarian sense in which it was doubtless intended, but it is *as* demonstrably false, if subjected to the rigorous interpretation which we ordinarily bestow upon the language wherewith science speaks. The ultimate *living* particle, whether it be the whole, the half, or the thousandth part of a single cell, must, of very necessity, be *all* living; the ultimate particle of *dead* matter must, of precisely parallel

* "Life, Matter, and Mind," page 33.

necessity, be *all* dead. Thus we are driven, by the inexorable force of logic, supported by a multitudinous array of facts, to the same conclusion which Beale long ago announced; namely, that matter exists in two diametrically opposite conditions, *living* and *dead;* that every organism, whether the humble ameba, or man himself, consists of matter in these two states. Thus far, the most censorious critic can find no fault with Beale, provided always he be a believer in the existence of a vital power or principle. But, to my mind, the *tests* by which this author proposes to demonstrate the presence or absence of life, and the conclusions to which a rigid belief in the infallibility of these tests necessarily force me, are alike unwelcome and unsatisfactory, and it is my purpose, in the present article, to show *why* this is so.

Dr. Beale's method of distinguishing, as between living and dead matter, and the sweeping conclusions which he draws therefrom, may be briefly formulated as follows:

*First:* The cell, wherever found, consists essentially of two parts; germinal matter (otherwise called cell contents, nucleus, protoplasm, bioplasm, endoplast), and formed material (otherwise called cell wall, periplast, intercellular substance, or products of secretion). The former may be distinguished from the latter by its capability of being stained by an ammoniacal solution of carmine, whereas the latter remains unaffected by the presence of the staining material.

*Secondly:* Masses of germinal matter, which readily take the carmine stain, are found more or less abundant in all tissues; in cartilage, in muscle, in nerve tissue, and in fibrous tissue; and its proportion to the whole mass is determined by the age of the animal, or by the amount of pabulum accessible thereto.

*Thirdly:* Germinal matter alone possesses the power of multiplication, of absorbing and converting nutriment, of building up and repairing the tissues, and of spontaneous movement; *therefore,* only germinal matter is *living* matter.

*Fourthly:* All matter which may not be colored by carmine, which may not grow and multiply, and which does not possess the power of movement, is no longer *living* but *dead* matter.

Here then we have the tests by which we are to distinguish living from dead matter: namely, the presence or absence of the capability of being stained by carmine, and the presence or absence of the power of multiplication and growth. Whether or not germinal or living matter may always and everywhere be stained by carmine, or whether or not it *never* stains by carmine, is a matter of utter indifference to us, only in so far as it enables us to distinguish between two kinds of matter. The mere fact that a certain part of the cell may be so colored does not of itself prove that it is either alive or dead. We may therefore dismiss this part of the subject with the remark that carmine furnishes us with a very convenient means of examining the different portions of the cell, and a probably reliable mode of distinguishing its living from its non-living elements; and with an equally convenient agent for the preparation of the solid tissues for microscopic study, although it is doubtless far less reliable as a test for the presence or absence of living matter in their substance.

Prof. Beale's second postulate demands a more extended consideration. No one who believes in vitality at all will dispute the statement that the power of self-multiplication and growth, together with the power of spontaneous movement, is satisfactory proof of the presence of life; no one will contend that the power of spontaneous movement or migration is absolutely necessary as a condition which must invariably accompany life. The real gist of the matter, then, lies in this question: is all matter necessarily dead which has not the power of increase or self-multiplication? To discuss this question at all satisfactorily it is necessary that we briefly recall the essential structure of the tissues. The solid structures, muscles, nerves, bones, ligaments, and cartilages—all the tissues of the body—are made up, in the adult, of minute masses of germinal or nuclear matter, and of larger masses of formed material or tissue proper. The former readily stains with carmine; is endowed with the power of converting pabulum into itself, and of converting itself into the latter; possesses, to a greater or less extent, the power of spontaneous movement, and sometimes of migration; and is, to all intents and purposes, indisputably *living* matter. The latter is essentially concerned in the execution of the duties for which it was created, or, as we usually and very correctly term it, *function;* it has no power to grow and increase itself of its own mere motion, even though it be abundantly supplied with the appropriate means of so doing; it does not readily become stained by carmine, and, when colored at all, the tint is pale and imperfect, and is readily removed; it sometimes possesses the power of spontaneous movement of a certain kind, sometimes does not; it never migrates from point to point, but is absolutely helpless when detached from its proper location in the body. Is it therefore *dead?* According to Dr. Beale, the absence of these peculiar endowments is competent proof of death; and this is simply equivalent to saying that at least seven-eighths of a great proportion of the tissues, which go to make up the body of an adult, do not enter upon the performance of their proper functions until after they have ceased to live. Dr. Beale fortifies himself in his position by citing the changes which occur in the cells of ordinary mildew, and in those of epithelial structures; to which we reply that, in those particular instances, and in others of like nature, he is, without any doubt, correct; but the further conclusion that, *therefore*, *all* formed material is lifeless matter, is not warranted. For this sweeping conclusion condemns us inevitably to the very disagreeable necessity of walking by means of dead muscles, of employing our several senses by means of dead nerves, of breathing by means of dead lungs, and, in fact, of performing all our various functions through the agency of structures which have absolutely passed the boundary between life and death. The following quotations will show clearly enough that I am not misrepresenting this author, or extorting from his language a meaning which he did not intend. "I apply the term *germinal matter* only to that which *lives*, *changes*, *converts*, *germinates*, *etc*. *Formed material*, on the other hand, never possesses any of these properties. It *has lived*, but is now *lifeless;* it may *be* changed, but it cannot change itself."* Again he says, more pointedly still, "the terms *living* and *dead* have for me a meaning somewhat different from that commonly accepted. If my arguments are sound, the greater part of the body of an adult man or animal, at any moment, consists of matter to all intents and purposes as dead as it would be if the individual itself were deprived of life. The formed material of the living cell is dead. The only part of the living cell and the living organism which is alive, is the germinal matter. Nothing can be regarded as alive or living but germinal matter in which vital changes alone take place."† Surely if all this be true,

* "How to Work with the Microscope," page 318.

† Op. cit., page 328.

the most case-hardened stoic may appropriately pray for delivery from " the body of this death."

For my own part, I am prepared to admit, and do fully believe, first, that the epithelial structures generally, the outer layer of mucous and serous membranes, the epidermic layer of the skin, the hair and nails, and the fluid portions (or " formed material ") of all secretions, of exudations and pus, consist of matter which is chiefly or wholly lifeless; more than that, I believe that in every truly living tissue, a considerable proportion of dead matter may always be found, by virtue of that retrograde metamorphosis which is the direct and inevitable result of use and wear, which is constantly going on from infancy to dotage, and which necessitates the provision of an extensive secretory apparatus for its immediate removal. Secondly, proceeding directly to the opposite extreme, we find matter (the so-called "germinal matter" or " bioplasm,") which is, in the highest sense, *living;* it inherently possesses the powers and properties already so frequently alluded to, namely, of growth, multiplication, and spontaneous movement. It is, without doubt, the ultimate repository of vital power—the absolute magazine of life. But between these two extremes; between matter absolutely dead on the one hand, and matter absolutely living, from Beale's standpoint, on the other hand, we find structures which belong neither to the one nor the other; structures which, deprived of some of the properties pertaining to the one, have not yet acquired some of the properties pertaining to the other; structures which, *wanting* the vital power necessary for *reproduction* and *growth*, *retain* so much of vital power as shall suffice for the performance of function; structures which, in passing over the boundary which separates germinal or growing matter from formed or "function-performing" material, carry with them just so much residual vital force as shall enable them efficiently and perfectly to perform their several functions, and maintain their own integrity while thus engaged.

Of this middle class, we may regard muscular tissue as the type, and I select it, because it is the most common, not because it is the only, type. It would be difficult for any man to convince himself, by any course of reasoning or experiment, that his muscles are composed, to the extent of forty-nine parts out of every fifty, of *dead* matter; and yet this is precisely what

Dr. Beale tells us. Lest the bare statement may not be deemed sufficient for those who have not access to his works, I quote his own words: "The contractile material of muscle may be shown to be continuous with the germinal matter, and oftentimes a thin filament of the transversely striated tissue may be detached with the oval mass of germinal matter still connected with it, showing that, as in tendon, the germinal matter passes uninterruptedly into the formed material. This contractile tissue is not, like the germinal matter which produced it, in a *living* state."*

Again, Prof. Beale divides the various movements which occur in living beings into two classes, as follows:

"I. Primary or Vital movements: affecting matter in the *living* state only, and occurring in every direction, as seen in the ameba, white blood corpuscle, and in germinal or living matter generally.

"II. Secondary movements, the *consequence* of vital movements, or of other phenomena, affecting matter which is *not* in a *living* state";† and in this latter class is placed ciliary movements, (as of ciliated epithelium,) muscular movements, (which he elsewhere calls a "physical" phenomenon,) molecular movements, and "movements of solid particles suspended in fluid in cells, caused by currents in the fluid." Quotations of similar import might be multiplied to an almost indefinite extent, from Prof. Beale's works, but these are sufficient for my present purpose. They show, conclusively enough, what, in the author's view, constitutes *death*, or dead matter; and, as a necessary corollary, that the various tissues only acquire their several *functional* powers after they have ceased to live. But, on page 311 of Prof. Beale's work on the Microscope, he gives a more formal statement of his views of the powers and properties of living matter, as follows:

1. The power of altering and appropriating certain soluble matters, and communicating to them properties or powers of the same nature as those which the living matter itself possesses.

2. The power of moving in all directions—the passage of one part of a living mass to another part, so that one portion may advance *itself* in front of another portion, or encircle another.

3. The power of causing the elements of matter to take up

* "Life, Matter and Mind," page 54.

† "How to Work with the Microscope," page 169.

definite relations towards one another, so that definite compounds, often exhibiting definite structures, may result, when the matter ceases to live.

4. The power of infinite increase.

Here, again, it will be observed that Prof. Beale carefully excludes several tissues upon which the various functions of the body fall, from the category of structures endowed with life.

If this doctrine be true, it must be regarded as an established fact, not only that we "die daily," but that we are constantly more dead than alive; that an exceedingly small proportion of the matter of which our several bodies is composed must necessarily be endowed with vital force sufficient to keep the other exceedingly large proportion in running order; in other words, that nature, reversing her ordinary laws, rules in this case by minorities. To what, then, is muscular contraction due? "To a 'disturbance' (electrical or otherwise) in the neighborhood of a contractile tissue," answers Dr. Beale. Why import the foreign element of "electrical disturbance," when the native element of vital force will answer, not only just as well, but a great deal better? Does nature ordinarily carry on her work by means of "disturbances, electrical or otherwise"? If the muscles which guide the pen with which these words are written are dead already, what change will they undergo at the moment when life shall altogether cease? He who accepts the theories of Beale must needs reply, "absolutely *no* change, except the death of their germinal matter." Then, surely, the same "disturbance" ought to produce the same result.

A careful consideration of this subject, will, I think, convince any one, save Dr. Beale himself, that the teachings of this most accomplished of microscopists are, on the point here discussed, essentially erroneous. No tissue can appropriately be called lifeless, while it is yet capable of performing its specific functions in that "living temple" whereof it forms a part. Dead as to the power of the conversion of pabulum; dead as to the power of reproduction; dead as to the power of traversing the field of the microscope by spontaneous movement of its component particles or of its entire mass, it indeed may be; but dead, in the ordinary sense of that fearful word, and in the sense in which it is constantly employed by Prof. Beale, it assuredly is *not*. Whether the life which pervades and animates the tissues be called a "functional" life, or by some other name, it matters not; whether the powers they manifest be attributed to an "electrical disturbance," or to some other cause equally far-fetched and equally absurd, it matters not; whether theorists continue cudgeling their brains to find explanation for that which needs no explanation, it matters not; but one thing is certain—that the great mass of scientific and thinking men will continue to believe that *all* the tissues of their bodies are under the constant guidance of that old-fashioned but unerring vital force which has already withstood ruder attacks than that of the ingenious writer and laborious investigator whose doctrines are here considered, and to whom we all owe so much.

